# Estimating cardiovascular health gains from eradicating indoor cold in Australia

**DOI:** 10.1186/s12940-022-00865-9

**Published:** 2022-05-17

**Authors:** Ankur Singh, Anja Mizdrak, Lyrian Daniel, Tony Blakely, Emma Baker, Ludmila Fleitas Alfonzo, Rebecca Bentley

**Affiliations:** 1 Centre for Epidemiology and Biostatistics, Melbourne School of Population and Global Health, University of Melbourne, Level 3, 207, Bouverie Street, Melbourne, Victoria 3010 Australia; 2 Department of Public Health, University of Otago, Wellington, New Zealand; 3 Australian Centre for Housing Research, The University of Adelaide, Adelaide, Australia; 4 Population Interventions Unit, Centre for Epidemiology and Biostatistics, Melbourne School of Population and Global Health, University of Melbourne, Melbourne, Australia; 5 Centre for Health Equity, Melbourne School of Population and Global Health, University of Melbourne, Melbourne, Australia; 6 Centre for Health Policy, Melbourne School of Population and Global Health, University of Melbourne, Melbourne, Australia

**Keywords:** Cardiovascular disease, CVD, Indoor cold, Policy simulation

## Abstract

**Background:**

Exposure to cold indoor temperature (< 18 degrees Celsius) increases cardiovascular disease (CVD) risk and has been identified by the WHO as a source of unhealthy housing. While warming homes has the potential to reduce CVD risk, the reduction in disease burden is not known. We simulated the population health gains from reduced CVD burden if the temperature in all Australian cold homes was permanently raised from their assumed average temperature of 16 degrees Celsius to 20 degrees Celsius.

**Methods:**

The health effect of eradicating cold housing through reductions in CVD was simulated using proportional multistate lifetable model. The model sourced CVD burden and epidemiological data from Australian and Global Burden of Disease studies. The prevalence of cold housing in Australia was estimated from the Australian Housing Conditions Survey. The effect of cold indoor temperature on blood pressure (and in turn stroke and coronary heart disease) was estimated from published research.

**Results:**

Eradication of exposure to indoor cold could achieve a gain of undiscounted one and a half weeks of additional health life per person alive in 2016 (base-year) in cold housing through CVD alone. This equates to 0.447 (uncertainty interval: 0.064, 1.34; 3% discount rate) HALYs per 1,000 persons over remainder of their lives through CVD reduction. Eight percent of the total health gains are achievable between 2016 and 2035. Although seemingly modest, the gains outperform currently recommended CVD interventions including persistent dietary advice for adults 5–9% 5 yr CVD risk (0.017 per 1000 people, UI: 0.01, 0.027) and persistent lifestyle program for adults 5–9% 5 yr CVD risk (0.024, UI: 0.01, 0.027).

**Conclusion:**

Cardiovascular health gains alone achievable through eradication of cold housing are comparable with real-life lifestyle and dietary interventions. The potential health gains are even greater given cold housing eradication will also improve respiratory and mental health in addition to cardiovascular disease.

**Supplementary Information:**

The online version contains supplementary material available at 10.1186/s12940-022-00865-9.

## Introduction

The World Health Organisation (WHO) Housing and Health guidelines make a strong recommendation that indoor temperatures should be above 18 degrees Celsius to protect residents from the harmful health effects of cold indoor environments [1]. Recent work suggests that many homes, even in the relatively mild or temperate climates of Australia, do not meet this standard [2, 3]. Cold indoor temperature is associated with elevated blood pressure and randomised controlled trials (RCTs) have shown that interventions increasing indoor temperatures reduce systolic blood pressure [4]. Observational studies, from the UK and 16 middle to high income countries also report consistent findings and confirm an association between low temperature and increased systolic blood pressure [5–7]. This means that intervening to improve the indoor temperature control should reduce cardiovascular disease incidence and prevalence.

Reducing exposure to unhealthy indoor temperature is achievable through interventions such as insulation, weatherization (draft-sealing), efficient heating/cooling appliances, cheaper fuel alternatives (solar panels), and subsidies and market-based initiatives [1]. Generating standardised estimates of the health gains from housing-focussed interventions such as these allows for direct comparison of their utility with other more commonly used public health interventions (e.g. tobacco control, pharmaceuticals). This paper aims to estimate the total potential cardiovascular health gains from a hypothetical ‘magic wand’ intervention that lifts all cold housing from an average indoor temperature during waking hours of 16 to 20 degrees Celsius in the six coldest months of the year.

Health gains from prevention occur many years into the future, requiring simulation modelling that quantifies health gain using summary measures such as health adjusted life years (HALYs). By using a measure such as a HALY, one can also compare the health impacts of otherwise disparate interventions [8, 9].

We used simulation modelling to quantify the health gain through a reduction in cardiovascular disease that could be achieved if exposure to indoor cold was hypothetically eliminated in three states of Australia [10]. Using an existing league table of preventive interventions for Australia and New Zealand [11], we examine the relative population health gains of hypothetical eradication of indoor cold with other actual prevention interventions. There has been limited simulation of future health benefits of reducing cold housing. For example, a NZ cost benefit analyses examined retrofitting insulation, but did not look at cold housing as its own entity, the total contribution (or ‘envelope’) of health gain from eradicating cold housing and did not use a structured disease/condition approach such as that used in burden of disease studies. Our paper applies a disease or condition-based approach, focusing on cardiovascular disease for which the evidence base is strongest (through changes in blood pressure) [12].

Methods.

### Intervention conceptualisation

We based our estimate of the effect of cold housing on cardiovascular disease burden on two sources of evidence. First, the negative effect of cold indoor temperature on blood pressure [4, 13]. Second, the effect of heightened blood pressure on cardiovascular disease risk (specifically ischemic heart disease and ischemic and haemorrhagic stroke) [14] (see supplementary file 1). We assume no time lag in response to temperature change.

### Model overview

We used a proportional multistate lifetable (pMSLT) simulation model to estimate health gains achievable through interventions on exposure to cold indoor temperature within a specified population [10]. We simulated the 2016 Australian population through to 2126 (maximum potential lifetime of the living cohort in 2016 being set to 110 year of age) in annual time steps with transition probabilities for all-cause mortality, and incidence and case fatality rates of cardiovascular diseases in subsidiary lifetables. This model was applied once for Business as Usual (BAU), based on the current prevalence of cold housing (prevalence assumed unchanging into the future), and then for the intervention (hypothetical elimination of cold housing) by altering the CVD incidence rates given the shift in population average blood pressure. The two components of the model are linked by population impact fractions (PIFs), that summarise the proportion reduction in diseases associated with change in indoor temperature exposure for cohorts defined by age and sex.

We modelled relevant cardiovascular diseases (heart disease and stroke) as independent of each other in parallel lifetables. Estimated changes in disease-specific morbidity and mortality rates were summed together in an overall lifetable at each annual cycle. The ‘health adjustment’ to convert life years gained to HALYs gained was achieved by subtracting off each life year gained the proportion ‘lost’ due to morbidity, using years of life lived with disability (YLDs) from burden of disease studies divided by the population in each sex by age-group as a measure of proportionate morbidity.

### Input parameters

We have presented data inputs with their sources in Table 1.

**Table 1:** Key data Inputs

Parameter	Data Source	Comments/ notes/ model and data assumptions	Value
Unhealthy indoor temperature prevalence at base year 2016	AHCD	Prevalence of people experiencing indoor cold temperature was obtained from the Australian Housing Conditions Dataset (AHCD) [15]. The AHCD survey asked participants ‘Are you able to warm your house during winters’? Those responding ‘No’ were considered as experiencing indoor cold temperature. The original question was derived from the English Housing Survey [17]. We accounted for age variations in the prevalence as estimated from the AHCD *Uncertainty*: Double of standard errors in age-specific prevalence obtained from AHCD with correlation of 1	5.74% (Refer to Table 2 for age variations)
Average temperature in cold houses		Average outdoor temperatures: Victoria (15.04 °C), New South Wales (18.43 °C), South Australia (20.19 °C) We assume average indoor cold temperature at 16 Celsius	
All-cause mortality rates	GBD	Data on all-cause mortality rates by sex and age group for 2016 were obtained from the Global Burden of disease results tool and inputted directly [28]	Refer to Table 2 for age and sex variations
All-cause morbidity rates	GBD	Data on years of life lived with disability (YLD) were obtained from the Global Burden of Disease study for each sex and age group in 2016. No time trend was allowed, as YLD rates by age in the GBD have not changed much over time. Morbidity rates were directly inputted in the main life table to estimate HALYs [28]	Refer to Table 2 for age and sex variations
Disease specific incidence, prevalence and case fatality rates	GBD	We applied national disease-specific estimates from GBD [28] to the population of three states New South Wales, Victoria and South Australia. Comparison of disease specific morbidity across the three states and national estimates showed a maximum of 10% difference – therefore we applied Australian disease data to these three states. The disease-specific incidence rates, prevalence and mortality rates, and case fatality rates (mortality rate divided by prevalence) for ischemic heart disease and stroke were obtained from the GBD data [28]. Stroke includes ischemic stroke and haemorrhagic stroke (subarachnoid and intracerebral). Disease specific rates for subarachnoid and intracerebral haemorrhagic stroke were summed and the ratio to ischemic stroke was included in the model for uncertainty analysis. All disease-specific epidemiological inputs were processed through DISMOD II and used to ensure coherence and smoothing for age [29] Annual Percentage Changes: the annual percentage changes were estimated using Poisson regression on incidence rates and case fatality rates from 1990 to 2016 GBD data and included as inputs to the PMSLT *Uncertainty:* ± *5% SD (log normal distribution for incidence), correlations 1.0 between sexes for all disease*	Refer to Table 2 for age and sex variations
Disease specific morbidity	IHME/GBD	The sex and age specific disability rates were calculated as disease’s YLD obtained from GBD [28]divided by the number of prevalent cases Uncertainty: ± 10% SD	
Relative risk from indoor cold to systolic blood pressure	Review of relative risks as part of the project	Using evidence and search terms from the WHO Housing and Health Guidelines, we reviewed the health effects of exposure to indoor cold. Our review found consistent evidence for the effect of indoor cold on hypertension. We performed risk of bias assessment using ROBINS-E and ROB tools on interventional and observational studies on the relationship between indoor cold and systolic blood pressure. Two studies (one cohort [6] and one randomised controlled trial[4]) were found to have low to moderate risk of bias. Relative risk from the randomised controlled trial was used *Uncertainty: As provided by* Saeki, Obayashi [4]	5.8 mmHg (95% CI (-9.3, -2.4)) More detailed review results presented in Table 1 in Appendices
Systolic blood pressure distribution	ABS	Data on systolic blood pressure by age and sex was obtained from the National Health Survey 2017–18 from the Australian Bureau of Statistics (ABS) [30]. Mean and standard deviations of systolic blood pressure were included as input to the pMSLT simulation model *Uncertainty: As provided by the National Health Survey* [30]	Refer to Table 4 in Appendices for age and sex variations
Relative risk from systolic blood pressure to ischemic heart disease and stroke	Forouzanfar, Liu [20]	Rate ratios for systolic blood pressure to ischemic heart disease, ischemic stroke and haemorrhagic stroke were taken from IHME GBD [28] *Uncertainty: As provided by Forouzanfar, Liu *[20]	Refer to Table 5 in Appendices for age variations

#### Base year and BAU parameters

Estimates of the number of people exposed to inadequate indoor temperature by age and sex was obtained from the Australian Housing Conditions Dataset (AHCD) survey [15, 16] and assumed to be constant into the future The AHCD survey asked participants ‘Are you able to warm your house during winters’? This question was derived from the English Housing Survey [17]. Those responding ‘No’ were considered as exposed to cold housing. We stratified the prevalence of exposure by age.

Data on the age and sex distribution of the Australian population was obtained from the Australian Population Census 2016. Disease-specific incidence, prevalence and case fatality rates were obtained from IHME Global Burden of Disease for Australia. We checked for coherence between epidemiological parameters derived from this array of data sources (i.e., incidence, case fatality and prevalence) for each disease by examining plotted trends and further processed them through epidemiological tool DISMOD II to use as data inputs [18].

### Intervention specification

Our intervention was to increase the average temperature in cold homes from 16 to 20 degrees Celsius. The relative risk of high blood pressure from exposure to indoor cold was estimated from a randomised controlled trial [4]. This study reported a difference of 5.8 mmHg (95% CI (-9.3, -2.4)) between an intervention group, who occupied a room heated to 22 °C, and the control group who occupied a room kept stable at 12 degrees Celsius. Both groups were given sufficient clothing and bedclothes to be warm. Both groups were exposed to the respective interventions for 11 h during the night and blood pressure was measured in 15 min intervals at night time and in the morning after rising. The 5.8 mmHg difference was estimated when awake (as there was no difference when asleep due to compensation using more bedding in the experimentally colder group). This effect estimate was converted into an absolute change achievable in systolic blood pressure per 1 degree Celsius temperature increase for the simulation model.

Data on the prevalence of people experiencing indoor cold was obtained from the AHCD that representatively sampled housing from 4,500 households' condition across three Australian States (Victoria, New South Wales and South Australia) [15]. The measure comprised a self-reported assessment of ability to keep warm indoors at home during cold winter weather. The average outdoor temperature for Victoria in 2016 was 15.0 degrees Celsius, for New South Wales was 18.4 degrees Celsius and for South Australia was 20.2 degrees Celsius [19]. To account for seasonal variations in temperature and time spent outside homes we assumed that our simulated population is exposed to indoor cold ranging from half (the awake 2/3rds of the day for the colder half of the year for those people at home most of the day and accounting for inadequate bedding) to one-sixth (same logic, but for people working or out of the home for approximately half of waking hours) of the time. A beta distribution for uncertainty in the intervention effect estimate was applied to account for variability in this exposure time (beta distribution Alpha 1: 10.5, Alpha 2: 22, median 31.9%, 2.5th percentile 17.6%, 97.5th percentile 49.0%). To simulate the effect of indoor cold on blood pressure we estimated the difference between cold houses (average temperature of 16 degrees Celsius) and adequately warmed houses (average temperature of 20 degrees Celsius). Assuming a short latency of cold to blood pressure, the change in average blood pressure across the year was calculated within each iteration of the simulation as: this proportion of the year exposed ranging from 1/6 to 1/2; multiplied by the difference in temperature (4 degrees, by lifting average cold housing from 16 to 20 degrees); multiplied by the RCT-based estimate of change in systolic blood pressure per 1 degree Celsius.

Relative risks for the causal relationship between systolic blood pressure and ischemic heart disease, ischemic stroke and haemorrhagic stroke were obtained from the Global Burden of Disease (GBD) study [20] (see supplementary Table 5). The intervention was simulated on the 2016 population for the same jurisdictions covered by the Australian Housing Conditions datasets (i.e., Victoria, New South Wales and South Australia). The intervention (eradication of cold housing) was modelled as lifelong. BAU exposure to cold housing in the future was based on exposures in 2016 for cohorts defined by age-group and sex.

### Analyses

Probabilistic uncertainty analyses using a Monte Carlo simulation method was conducted on input parameters (see Table 1) [10]. More generous uncertainty was applied where we were less confident on input parameters (for example the subjective measurement of indoor cold in Australia). Simulations were run using the ERSATZ add in to Excel with 2000 iterations used to generate 95% uncertainty intervals (UI) for the HALY estimates.

Our outputs included HALYs gained by indoor cold eradication within a life-time and ten- and -twenty-year time horizons. Outcomes were reported both with 0% and 3% discount rates and also per 1000 persons alive in 2016.

We compared the estimated HALYs gained from cold housing eradication with other CVD-related interventions described in interactive league tables [11].

## Results

Nearly 6% of the population were estimated to experience cold housing. Younger people reported a higher prevalence of indoor cold exposure (9% under the age of 30 years) compared to older ages (3.4% over the age of 75 years) (Table 2).

**Table 2:** Input data for starting cohort in 2016

Age group	n	Indoor cold	All cause		Systolic BP	Ischemic heart disease			Ischemic Stroke			Hemorrhagic Stroke		
		Prevalence (%)	Mortality rate	Morbiity (proportion reduction in life year for HALY)	Mean (SD)	IR	CFR	DR	Incidence	CFR	DR	IR	CFR	DR
Males														
0–4	514,007	8.98	18	0.032		0	0.000	0	5	0.000	23	2	0.001	0
5–9	500,318	8.98	9	0.039		0	0.000	0	5	0.001	52	2	0.002	0
10–14	462,301	8.98	11	0.056		0	0.000	0	5	0.003	81	2	0.003	0
15–19	478,494	8.98	42	0.082	119.5 (23.7)	1	0.002	0	5	0.004	110	2	0.005	0
20–24	552,493	8.98	64	0.098	119.5 (23.7)	2	0.011	2	6	0.005	143	3	0.007	0
25–29	578,722	8.98	75	0.107	120.3 (16.9)	5	0.017	3	8	0.005	191	5	0.009	0
30–34	571,793	8.98	93	0.114	120.3 (16.9)	15	0.024	5	14	0.007	266	8	0.012	1
35–39	516,034	7.49	118	0.121	121.3 (16.0)	38	0.028	10	24	0.008	383	13	0.015	2
40–44	512,660	7.49	169	0.127	121.3 (16.0)	86	0.027	20	39	0.009	573	20	0.017	3
45–49	499,837	7.49	237	0.134	126.5 (15.6)	177	0.024	38	60	0.009	885	28	0.019	5
50–54	486,978	7.49	354	0.145	126.5 (15.6)	312	0.021	63	90	0.009	1323	37	0.022	8
55–59	465,154	5.52	535	0.159	132.4 (18.5)	486	0.018	97	134	0.010	1949	48	0.027	12
60–64	411,857	5.52	800	0.177	132.4 (18.5)	721	0.017	154	200	0.011	2775	62	0.037	19
65–69	377,806	5.52	1227	0.201	134.9 (16.3)	1001	0.017	255	286	0.014	3874	81	0.060	30
70–74	331,006	5.52	2018	0.230	134.9 (16.3)	1310	0.018	376	386	0.018	5774	105	0.107	50
75–79	205,258	3.40	3462	0.260	136.5 (16.0)	1696	0.023	501	518	0.026	8841	145	0.224	95
80–84	137,498	3.40	6308	0.297	136.5 (16.0)	2245	0.035	649	727	0.043	12,677	217	0.429	178
85–89	84,731	3.40	11,646	0.342	140.2 (15.7)	3096	0.066	770	1119	0.076	16,667	350	0.720	331
90–94	32,443	3.40	18,487	0.388	140.2 (15.7)	4561	0.151	817	1687	0.139	19,947	536	1.085	533
95–99	6255	3.40	30,624	0.429	140.2 (15.7)	5678	0.224	813	2077	0.187	22,295	623	1.254	622
100–104	590	3.40	30,624	0.429	140.2 (15.7)	5678	0.224	813	2077	0.187	22,295	623	1.254	622
Females														
0–4	486,858	8.98	15	0.028		0	0.000	0	6	0.000	37	3	0.002	0
5–9	475,040	8.98	8	0.038		0	0.000	0	6	0.000	85	3	0.002	0
10–14	437,256	8.98	9	0.058		0	0.000	0	5	0.002	129	3	0.002	0
15–19	455,281	8.98	23	0.096	107.6 (16.5)	1	0.001	0	5	0.004	171	3	0.003	0
20–24	528,234	8.98	23	0.117	107.6 (16.5)	1	0.004	1	7	0.004	219	4	0.003	0
25–29	579,388	8.98	29	0.125	108.5 (17.1)	2	0.006	3	10	0.003	293	6	0.005	0
30–34	580,157	8.98	39	0.130	108.5 (17.1)	4	0.008	5	17	0.004	387	11	0.007	1
35–39	518,900	7.49	65	0.137	112.5 (16.2)	8	0.011	9	27	0.005	553	17	0.010	2
40–44	522,574	7.49	91	0.145	112.5 (16.2)	17	0.012	17	41	0.006	789	26	0.011	3
45–49	521,716	7.49	141	0.152	119.6 (20.1)	37	0.012	28	59	0.006	1146	35	0.012	5
50–54	502,591	7.49	214	0.156	119.6 (20.1)	68	0.011	44	82	0.007	1653	46	0.012	7
55–59	485,496	5.52	311	0.164	126.8 (19.0)	114	0.011	65	112	0.007	2375	57	0.013	9
60–64	433,268	5.52	462	0.178	126.8 (19.0)	189	0.012	97	163	0.007	3300	70	0.016	13
65–69	392,690	5.52	721	0.200	133.6 (16.7)	294	0.013	161	240	0.009	4412	87	0.023	21
70–74	300,714	5.52	1233	0.224	133.6 (16.7)	427	0.016	250	337	0.012	6132	111	0.042	36
75–79	229,357	3.40	2242	0.249	137.9 (14.8)	610	0.024	364	460	0.020	8737	153	0.103	75
80–84	173,515	3.40	4361	0.289	137.9 (14.8)	889	0.040	514	664	0.038	12,137	229	0.232	153
85–89	127,250	3.40	8819	0.337	140.8 (16.2)	1396	0.081	695	1085	0.073	16,308	368	0.446	312
90–94	64,694	3.40	16,373	0.386	140.8 (16.2)	2607	0.192	802	1728	0.148	20,097	562	0.735	547
95–99	16,786	3.40	30,867	0.434	140.8 (16.2)	3630	0.289	833	2178	0.208	22,859	652	0.869	652
100–104	2018	3.40	30,867	0.434	140.8 (16.2)	3630	0.289	833	2178	0.208	22,859	652	0.869	652

IR, DR and Mortality rate presented per 100,000 persons and CFR per person

*IR* Incidence Rate, *CFR* Case Fatality Rate; DR: Disability rate

We estimated that cold eradication generated an additional 1.64 (95% uncertainty interval (UI): 0.232, 4.90) undiscounted HALYs per 1000 persons compared with BAU across the lifespan and 0.447 (95% UI: 0.064, 1.34) with a 3 per cent discount applied (Table 3). From the perspective of the ‘target population’ (i.e. the 5.74% in cold housing) this equates to 29 undiscounted HALYs per 1000 people (i.e. 1.64/0.0574), or an average (across ages) of one and a half weeks of additional health life per person in cold housing through CVD alone. Eight per cent of this gain is achieved within the first 20 years: we estimated 0.135 (UI: 0.019, 0.442) undiscounted HALYs per 1000 persons between 2016 and 2036. We note that at this point, most cohort members have not reached the ages of high CVD risk.

**Table 3:** Future HALYs per people under BAU and HALYs gained by eradication of unhealthy indoor temperature

	Population in 2016	Business as usual			Incremental gains in HALYs compared to BAU, for elimination of cold indoor temperature (95% uncertainty interval in parentheses)					
		Undiscounted			Undiscounted			Discounted at 3%		
		Lifetime	2016–2025	2016–2035	Lifetime	2016–2025	2016–2035	Lifetime	2016–2025	2016–2035
Total	15,560,018	589,561,555	128,591,729	243,278,999	25,500 (3,610; 76,200)	469 (67; 1480)	2,110 (302; 6,560)	5,240 (995; 20,800)	388 (56; 1220)	1,432 (206; 4,460)
Per 1000 persons		37,889.52	8264.24	15,634.88	1.64 (0.232; 4.90)	0.030 (0.004; 0.095)	0.135 (0.019; 0.422)	0.447 (0.064; 1.34)	0.019 (0.004; 0.079)	0.092 (0.013; 0.287)

All numbers rounded to three meaningful digits, with a maximum of three decimal places

**Fig. 1:**
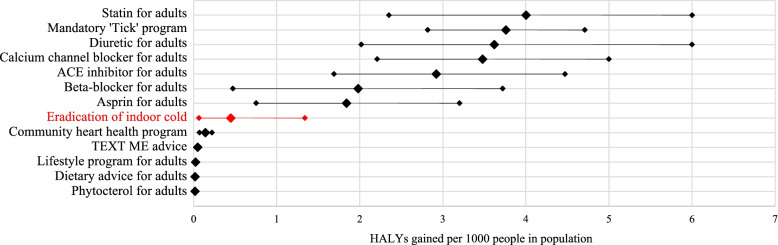
Ranking of eradicating exposure to indoor cold compared to actual preventive interventions. Note: All interventions in adults aged 35–84 years old with lifetime duration unless otherwise stated in the footnotes. Standard dose of statin for adults with 5–9% five-year risk of CVD through primary care practice. Mandataory ‘Tick’ program to reduce salt in bread, margarine and breakfast cereal by 10.65 mgNa day for men and 7.3 mgNa for women with at least 5% five-year risk of CVD. Standard dose of diuretic for adults with 5–9% five-year risk of CVD through primary care practice. Standard dose of calcium channel blocker for adults with 5–9% five-year risk of CVD through primary care practice. Standard dose of ACE inhibitor for adults with 10–14% five-year of CVD risk through primary care practice. Standard dose of beta-blocker for adults with 5–9% five-year CVD risk through primary care practice. Standard dose of aspirin for adults with 5–9% five-year risk of CVD through a combination of primary care practice prescription (50%) and over the counter (50%) use. Community heart health program to promote dietary change, physical activity and smoking cessation for the whole population. Tobacco, Exercise and Diet Messages (TEXT ME) providing advice, motivation, information and support to improve health related behaviours plus usual care, for individuals with documented CHD over a lifetime. Lifestyle program providing professional advice in diet and physical activity for adults with 5–9% five-year risk of CVD. Dietary advice for adults with 5–9% five-year CVD risk. Phytosterol-enriched margarine (92 g per kg of margarine) for adults with 5–9% five-year risk of CVD [31]

In Fig. 1 we have compared the quantified discounted HALYs gained per 1000 persons with actual CVD interventions in the Australia New Zealand Health Intervention League Table. The hypothetical eradication of indoor cold (HALYs gained during lifetime: 0.447 (UI: 0.064, 1.34) outperformed many CVD interventions with a lifetime horizon for accrual of health gains. For example, a persistent dietary advice intervention for adults with 5–9% 5 yr CVD risk (0.017, UI: 0.01, 0.027), a persistent lifestyle program taken up by adults with 5–9% 5 yr CVD risk (0.024, UI: 0.01, 0.027) and persistent Community Heart Health Program (0.141, UI: 0.071, 0.221).

## Discussion

This is the first study that quantifies cardiovascular disease related health gains from raising the temperature in Australian cold housing by 4 degrees Celsius. We estimate these health gains are on par with currently recommended lifestyle interventions to reduce CVD risk including dietary advice and lifestyle and community targeted programs. Further, we note that this is only part of the intervention’s total health benefit with gains from reductions in respiratory and mental ill-health also likely to follow interventions that warm the indoor temperature of cold homes. Our study should be viewed as a departure point for estimating actual interventions (e.g., retrofitting houses) and our findings support and extend previous research on the cost effectiveness of installing insulation, for example, to improve occupant’s health in New Zealand [12].

Our study has several limitations that should be acknowledged. First, there is ‘structural’ or mechanistic uncertainty in how changes in indoor temperature flow to changes in blood pressure and then onto CVD incidence. We have used the effect size of cold housing onto blood pressure from a well-conducted randomised controlled trial that examined a short-term effect of temperature change on blood pressure. However, this is essentially the effect of temperature on *labile* hypertension (immediate fluctuations in blood pressure) rather than longer-term average blood pressure. By using this effect size in our simulation models, we are assuming that the cumulative impact of labile hypertension has the same impact as a constant but lesser increased in blood pressure (i.e. a cold housing impact of raising systolic blood pressure by 2 mm Hg during waking hours whilst in your house in winter is assumed to be similar to a 0.33 (1/6^th^) to 1 (1/2) mmHg increase in blood pressure over the whole year). There is some literature suggesting poor cardiovascular effects of labile hypertension [21, 22]. Second, a simulation study forces one to pull together all the data inputs necessary to quantify impacts, and often discloses data weaknesses. In conducting this study, we found that the underlying evidence on the quantitative association of indoor temperature with health outcomes is lacking (e.g. we could not find robust estimates of cold housing impacts on respiratory disease incidence or severity, restricting us to a focus on blood pressure as a mediating factor to CVD). Third, as highlighted at the beginning of this discussion, our estimates for health gains are conservative because they only include cardiovascular disease related health impacts. Future modelling may quantify population health gains achievable through reduction of morbidity and mortality across a greater range of diseases as well as changes in disease-specific incidence rates. Fourth, there is no quality objective data (to our knowledge) on the exact proportion of houses in Australia that have an indoor air temperature in living areas less than the recommended threshold of 18 degrees Celsius (and for what duration of the year, and other aspects of exposure characterisation). Instead, we had to use subjective measures of indoor temperature from the AHCD and ‘crosswalk’ this to being equivalent to the proportion of houses that are cold. Finally, we assumed no time lags between indoor temperature change and change in incidence in cardiovascular diseases as blood pressure responds immediately to temperature, and CVD rates change quickly (within a couple of years) to changes in blood pressure.

Inadequate housing is strongly patterned by socioeconomic position [23–26], and provides a domain for intervening to both improve health and to reduce inequalities in health. Next steps in research in this field include equity-informed estimation of cost effectiveness of remedying cold housing through actual interventions, from both a health system and a wider societal perspective given that housing interventions also usually lead to other social impacts such as less energy consumption (see Chapman, Howden-Chapman [27] for an early example). Specific interventions to evaluate include insulation, weatherization (draft-sealing), efficient heating/cooling appliances, cheaper fuel alternatives (solar panels), and subsidy and market-based initiatives to achieve reduction of indoor cold. From the point of view of CVD-reduction specifically, we note that existing interventions mainly involve behavioural or pharmaceutical treatments. Through this modelling we were able to compare health gains due to the eradication of indoor cold with existing cardiovascular interventions and found that the benefits are on par, which creates opportunities to compare population health gains from housing interventions to more traditional medicalised or health behaviour interventions.

## Conclusion

This simulation modelling extends the current knowledge on ill health effects of indoor cold [1], but also reveals profound limitations in current knowledge of cold housing’s impact on health. Existing interventional studies from New Zealand also point to cost-effectiveness and equitable health benefits of retrofitting insulation to reduce indoor cold [12]. Our study straddles comparative risk assessment and intervention modelling, to create a strong policy argument for addressing indoor temperature. Our finding that substantial prospective population health gains are achievable by eliminating indoor cold in Australia is an important first step in estimating which interventions are the most cost-effective to deliver this. Moreover, this may be as effective in preventing and treating cardiovascular disease as some more medically focussed current approaches.

## Supplementary Information

**Additional file 1.**

## Data Availability

Data generated or analysed during this study are included in this published article and in its supplementary files. Unit record data from the Australian Housing Conditions Dataset is available from https://dataverse.ada.edu.au/dataset.xhtml?persistentId=doi:10.26193/RDMRD3

## Abbreviations

BAU: Business as Usual
CVD: Cardiovascular disease
GBD: Global Burden of Disease
HALYs: Health adjusted life years
PIFs: Population impact fractions
pMSLT: Proportional multistate lifetable
RCTs: Randomised controlled trials
UI: Uncertainty intervals
WHO: World Health Organisation
YLDs: Years of life lived with disability
