# Supplementary material for: Estimating cardiovascular health gains from eradicating indoor cold in Australia
Source: Environ Health. 2022 May 17;21:54. doi: 10.1186/s12940-022-00865-9 (PMC9112519; doi:10.1186/s12940-022-00865-9)
Supplement: Supplementary file 1 — Additional file 1. [file 12940_2022_865_MOESM1_ESM.docx]

Appendices

Project funded by Affordable Housing Hallmark Initiative seed funding

Contents

[**Supplementary file 1. Brief overview of literature on cold indoor temperature and health** 2](#_Toc83645693)

[Search terms 2](#_Toc83645694)

[Characterization of exposure to indoor cold 2](#_Toc83645695)

[Risk of bias assessment 2](#_Toc83645696)

[Table 1. Summary characteristics of included studies 3](#_Toc83645697)

[Table 2. Summary characteristics of the association between indoor cold and SBP 5](#_Toc83645698)

[**Measurement of Exposure(s) in Australia** 8](#_Toc83645699)

[Indoor temperature data in Australia 8](#_Toc83645700)

[Table 3. Summary characteristics of available datasets 8](#_Toc83645701)

[Gaps/inconsistencies in evidence 9](#_Toc83645702)

[**Data inputs into the model** 10](#_Toc83645703)

[Source of demographic data 10](#_Toc83645704)

[Population distribution by age and sex 10](#_Toc83645705)

[Source of data on disease burden 10](#_Toc83645706)

[All-cause mortality and morbidity rates 10](#_Toc83645707)

[Disease-specific incidence, prevalence, and mortality rates 10](#_Toc83645708)

[Disease-specific case fatality rates 10](#_Toc83645709)

[Disease-specific disability rates 10](#_Toc83645710)

[Disease-specific incident rate ratios by temperature status 10](#_Toc83645711)

[Source of data on exposure 12](#_Toc83645712)

[Prevalence of unhealthy indoor temperature in base year 12](#_Toc83645713)

[Trends in prevalence of unhealthy indoor temperature for projections 12](#_Toc83645714)

[Disease trends: Annual percentage change for all-cause mortality rates 12](#_Toc83645715)

[**DISMOD coherence checks on epidemiological data from IHME** 13](#_Toc83645716)

[Ischemic heart disease 13](#_Toc83645717)

[Ischemic stroke 14](#_Toc83645718)

[Haemorrhagic stroke 15](#_Toc83645719)

[References 16](#_Toc83645720)

**Supplementary file 1. Brief overview of literature on cold indoor temperature and health**

As part of the World Health Organization’s (WHO) housing and health guidelines, two separate systematic reviews were conducted to summarise health effects of low indoor temperature and high indoor temperature. Lower (unhealthy) indoor temperature was considered below 18 °C and higher (unhealthy) above 24 °C based on a previous conclusion of a WHO working group on indoor environment:

“there is no demonstrable risk to human health of healthy sedentary people living in air temperature of between 18 and 24 °C”

Search terms

A search was conducted on Medline using PubMed platform on 10 April 2020 to identify relevant studies without any date or language restrictions and using the following search strategy:

(Cold OR Cool OR Chill OR Low OR Minimum OR Severe OR Hibernal OR Hiemal) AND (Indoor OR Home OR Domestic OR Dwelling OR House OR Inside OR Household OR Outdoor OR Ambient OR Outside OR Exterior OR Threshold) AND (temperature OR climate OR thermal OR degrees OR weather) AND (“infant mortality” OR cardiovascular OR respiratory OR hospitalization OR admissions OR depression OR mood)

An additional search was conducted on 16 June 2020 with PubMed MeSH terms for indoor temperature, pulmonary and cardiovascular diseases.

Articles including measures of indoor temperature and its association with any health outcomes (respiratory diseases, cardiovascular diseases, BMI, psychological distress, etc) were included in the review.

Characterization of exposure to indoor cold

We identified 14 studies looking on the association between indoor cold and health outcomes, A summary of the evidence is presented on Table 1. Studies were conducted on adult populations from Shanghai (China), Aberdeen City, Scotland, New Zealand, North Staffordshire, the United Kingdom, Japan, Nara, Kansai Region, Japan, England, the United Kingdom.

The health outcomes investigated were. COPD, Respiratory symptoms, Asthma, URTI, Ambulatory blood pressure, BP 2hr prior to going to bed, mean BP at night, and BP 2hr after rising, Biomarkers (sBP, dBP, Mean arterial pressure, Total Blood Cholesterol, LDL, Insulin like growth factor, Hb, WBC). Most studies focused on the association between indoor cold and cardiovascular diseases. Therefore, we decided to focus on systolic blood pressure changes related to indoor cold.

Risk of bias assessment

A total of 8 studies were selected for risk of bias assessment (see Table 2). For the risk of bias assessment, we used RoB 2 for assessing randomised control trials (Saeki 2013, Saeki 2015). The ROBINS-E tool was then used for observational studies.

The overall quality of studies on indoor cold and cardiovascular diseases was poor, especially in the selection and adjustment for confounding factors. Two high quality papers were identified, from which we selected estimates of Shiue 2016, a cross-sectional study based on the UK for its similarities with the Australian population.

Table 1. Summary characteristics of included studies

| Exposure | Outcome | | Study | Design | Effect estimate |
| --- | --- | --- | --- | --- | --- |
| Living room temperature of 24°C for ≥two hours | Blood pressure | | Saeki 2015 (1) | RCT | Participants on living room temperature of >24°C had a 4.43mmHg decrease on SBP and DBP decreased by 2.33mmHg |
| 1°C increase in room temperature below the thresholds of 9°C and 12°C | Lung function of children with asthma | | Pierse et al. 2013 (2) | RCT | Temperatures <12° C had the greatest effect on lung function. For each 1° C increase in temperatures below 12° C, FEV improved by 10.06 mL (morning measure). |
| Indoor temperature below 18°C /18.2°C | Lung biomarkers/ Respiratory problems. | | Shiue 2016 (3)/ Mu et al. 2017 (4) 2017 | Cross-sectional/Cohort | The differences between ≥18° C vs <18° C were:  -Forced vital capacity of -0.04 L (95%CI -0.05; -0.03)  -Forced expiratory flow of -0.03 L (95%CI-0.04; -0.02)  -Peak expiratory flow of -0.06 L (95%CI -0.09; -0.04)  Shiue 2016  Indoor temperature of above 18.2°C slightly reduced respiratory problems (OR: 0.977; 95% CI: 0.962, 0.999)  Mu 2017 |
| Indoor temperature below 18°C | Blood biomarkers | | Shiue 2016 (3) | Cross-sectional | The differences between ≥18° C vs <18° C were:  -For SBP -0.67 (95%CI -0.72; -0.50)  -For DBP -0.61 (95%CI -0.72; -0.50) |
| More hours of indoor warmth at and above 21°C | Respiratory problems | | Osman et al. 2008 (5) | Cross-sectional | Spending at least 9 hours at ≥21°C living room temperature was associated with worse respiratory symptoms (−1.12 decrease in St George’s Respiratory score) |
| Rooms at 12°C compared to 22°C | Blood pressure | | Saeki et al 2013 (6) | RCT | There was a difference of morning measures of -5.8mmHg for sBP and -5.1mmHg for dBP for those at room temperature of 22°C when compared with participants on rooms at 12°C |
| Exposure | **Outcome** | **Study** | | **Design** | **Effect estimate** |
| Each 1°C increase | Blood pressure | | Bruce et al. 1991 (7)/ Barnett 2007 (8)/Zhao 2019(9) | Cross-sectional/  Cohort/Cross-sectional | An increase of 1°C in room temperature had an equivalent effect of 0.5 mmHg decrease in sBP and dBP.  Bruce 1991  An increase of 1°C of indoor temperature was associated to -0.31 mmHg reduction of SBP (95% CI: -0.44, -0.19)  Barnett (2007)  An increase of 1°C in room temperature was associated with -0.48 mmHg of SBP (95%CI: -0.72, -0.25) and -0.45 of DBP (95%CI: -0.63, -0.27)  Zhao (2019) |
| Each 1°C decrease | Blood pressure | | Saeki et al 2014 (10, 11)/ Woodhouse, Khaw, and Plummer 1993 (12) | Cohort | A 1°C decrease was associated to:  -An increase of 0.22mmHg in daytime sBP (95%CI:0.003, 0.43)  -A 0.33mmHg (95%Ci 0.11, 0.55) increase in sleep-through morning blood pressure  -A 0.31mmHg increase in pre-walking morning blood pressure (95% CI: 0.10, 0.52)  K Saeki et al. (2014); Keigo Saeki et al. (2014)  A 1°C decrease in room temperature was associated to a 0.9 mmHg increase in SBP (95%CI: 0.4,1.4) and 0.4 mmHg increase in DBP (95%CI: 0.2,0.7)  Woodhouse, Khaw, and Plummer (1993) |
| Mean difference in indoor temperature | Upper respiratory tract infection (URTI) | | Ross, Collins and Sanders 1990 (13) | Case-control | Bedrooms of children with URTI tended to be cooler overnight with a mean difference of -0.9° C |

Table 2. Summary characteristics of the association between indoor cold and SBP

| Study | Country | Design | Sample size | Population | Exposure | Outcome | Confounding factors | Measure of effect | Effect est. (95% CI) | Risk of bias |
| --- | --- | --- | --- | --- | --- | --- | --- | --- | --- | --- |
| Zhao 2019 (9) | UK | Cross-sectional | 4659 | People aged 16 years and over | 1°C increase | SBP | Age, sex, marital status, ethnicity, education, income, area level deprivation, BMI, GHQ-12, type 2 diabetes diagnosis, total cholesterol, sodium excretion, alcohol consumption, smoking status, physical activity level and outdoor temperature | Mean change (mmHg) | -0.48 (-0.72,  -0.25) | Serious |
| Shiue 2016 (3) | UK | Cross-sectional | 7997 | Adults aged 50 years and over | <18° C | SBP | Sex, age, previous blood pressure, dominant hand, height, weight, waist | Mean change (mmHg) | -0.67 (-0.72,  -0.5) | Moderate |
| Study | **Country** | **Design** | **Sample size** | **Population** | **Exposure** | **Outcome** | **Confounding factors** | **Measure of effect** | **Effect est. (95% CI)** | **Risk of bias** |
| Saeki 2015 (1) | Japan | RCT | 359 | Adults aged 60 years and over | >24°C | SBP | Age, sex, BMI, daily alcohol intake, current smoking, hypertension medication, diabetes, and physical activity, outdoor activity, and household income | Mean change (mmHg) | -4.43 (-7.88,  -0.97) | High |
| Saeki 2014 (11) | Japan | Prospective cohort | 880 | Adults aged 60 years and over | 1° C decrease | Daytime and night-time SBP | Age, sex, BMI, smoking, drinking, diabetes, calcium channel blocker, angiotensin-converting enzyme/ARBs, angiotensin receptor blockers, other  antihypertensi-ves, evening administration, and physical activity at BP measurement | Mean change (mmHg) | *Daytime SBP:*  0.22 (0.003, 0.43)  *Night-time SBP: -*0.05  (-0.24, 0.14) | Moderate |
| Study | **Country** | **Design** | **Sample size** | **Population** | **Exposure** | **Outcome** | **Confounding factors** | **Measure of effect** | **Effect est. (95% CI)** | **Risk of bias** |
| Saeki 2013 (6) | Japan | RCT | 146 | Adults aged 60 years and over | 22° C vs 12° C | Morning, night-time, and evening SBP | Age, gender, BMI and current smoking status | Mean difference (mmHg) | *Morning SBP:*  -5.8 (-9.3, -2.4)  *Night-time SBP:* -0.3  (-3.5, 2.8)  *Evening SBP:*  -5.1 (-8.9, -1.3) | Low |
| Barnett 2007 (14) | 16 middle to high-income count-ries | Prospective cohort | 1154344 | Adults aged 35-64 years | 1°C increase | SBP | Age, sex, and BMI | Mean change (mmHg) | -0.31 (-0.44,  -0.19) | Serious |
| Woodhouse 1993 (12) | UK | Prospective cohort | 96 | Adults aged 65-74 years | 1° C decrease | SBP | Age, sex, seasonal effect and mean daily outdoor temperature | Mean change (mmHg) | 0.9 (0.4, 1.4) | Serious |
| Bruce 1991 (7) | UK | Cross-sectional | 10345 | Adults aged 45-59 years | 1° C increase | SBP | Age and town | Mean change (mmHg) | -0.01 (-0.284, 0.264) | Serious |

**Measurement of Exposure(s) in Australia**

Indoor temperature data in Australia

Table 3. Summary characteristics of available datasets

| Study | Dataset | Sample | Characterisation of indoor temperature | Year/s |
| --- | --- | --- | --- | --- |
| Harrington, Aye (15) | Collected by authors | 273 households from the following locations: Cairns area (n=23), Brisbane metropolitan area (n=32), Byron Bay area (n=11), Sydney metropolitan area (n=71), West Gippsland area (n=72), Melbourne metropolitan area (n=52), other locations (n=4) | Average indoor temperature (day, month, time of day) | 2011 to 2014 |
| Ambrose, James (16) | Evaluation of the 5-star energy efficiency standard for residential buildings. (CSIRO report) | 414 houses from the following areas: Melbourne, Adelaide and Brisbane | Average indoor temperature.  Cooling and heating use. | June 2012 to Feb 2013 |
| Cheng, Galbally (17) | Indoor air project. The Centre for Australian Weather and Climate Research and Australian Bureau of Meteorology | 39 households from Melbourne, Victoria | Indoor temperature according to time of the day. | 2008 |
| Soebarto, Bennetts (18) | Collected by authors | 10 low energy apartments 8 km North East of Adelaide CBD | Indoor (living room) temperature was recorded in 15-minutes intervals | Jan to March of 2012 |
| Asumadu-Sakyi, Barnett (19) | Collected by authors | 77 houses located in Brisbane | Indoor temperature (living room) was recorded in 30 minutes intervals. | May 2017 to May 2018 |
| Daniel, Williamson (20) | Collected by authors | 40 houses in Darwin and Melbourne | Average temperature | Late 2012 to early 2013 |
| de Dear, Kim (21) | Collected by authors | 42 homes equipped with at least one A/C unit in Sydney and Wollongong | Indoor temperature at 15 minutes intervals. | March 2012 to March 2014 |
| Haddad, Pignatta (22) | Collected by authors | 106 low income households from NSW | Indoor temperature, air humidity and carbon dioxide in 30 min intervals | May 2018 to Feb 2019 |
| Study | **Dataset** | **Sample** | **Characterisation of indoor temperature** | **Year/s** |
| Loughnan, Carroll (23) | Collected by authors | 20 households from regional Victoria | Average indoor temperature | Feb to March 2012 |
| Baker, Daniel (24) | The Australian housing conditions dataset (AHCD) | 4501 households across South Australia, Victoria, and New South Wales | Survey respondents reported their ability to keep their houses warm in winter | Aug to Oct 2016 |

Gaps/inconsistencies in evidence

It is unclear how the sample sizes were estimated for most studies. Measures of indoor temperature were recorded in 10 to 30 minutes intervals and characterised as average day temperature, average month temperature and temperature according time of the day. Most measures were taken in the living area of the household. One study, AHCD, measured participant’s perception of indoor cold.

**Data inputs into the model**

Source of demographic data

Population distribution by age and sex

Data on population distribution was extracted from the Australian Bureau of Statistics 2016 census estimates for each State (NSW, SA, and VIC).

Source of data on disease burden

All-cause mortality and morbidity rates

Data on all-cause mortality and morbidity rates were obtained from the Global Burden of Disease results tool and directly entered the model.

Disease-specific incidence, prevalence, and mortality rates

Australia wide 2016 disease-specific incidence and prevalence rates per 100.000 by sex and 5-year age groups were obtained from GBD results tool for Ischemic heart disease, ischemic stroke, subarachnoid haemorrhage, and intracerebral haemorrhage. GBD estimates for subarachnoid haemorrhage and intracerebral haemorrhage were added together to calculate the incidence, mortality, and prevalence rates of haemorrhagic stroke. Incidence, prevalence and mortality rates per-person for ischemic heart disease, ischemic stroke and haemorrhagic stroke were used as inputs to DISMOD II.

Disease-specific case fatality rates

Case fatality rates were calculated for each of the three diseases using the following formula:

Case Fatality Rate in year (x)=

Death rate attributed to disease in year (x)/ prevalence of disease in year (x)

Estimated case fatality rates for 2016 were used as inputs to DISMOD II.

Disease-specific disability rates

To estimate disability rates for each of the three diseases YLD number by sex and 5-year age groups for ischemic heart disease and ischemic stroke, subarachnoid haemorrhage and intracerebral haemorrhage were extracted from GBD results tool. We then estimated YLD rates for haemorrhagic stroke by adding YLDs number for subarachnoid and intracerebral haemorrhage. Finally, disability rates by sex and 5-year age groups were calculated as follows:

Disability Rate in year (x) =

YLD in year (x) / (Population size in year (x) x dismoded prevalence of disease in year (x))

*Table 4. Systolic blood pressure distribution, ABS*

| Males |  |  |
| --- | --- | --- |
| Age group | **Average SBP** | **Std Dev** |
| 18–24 | 119.5 | 23.7 |
| 25–34 | 120.3 | 16.9 |
| 35–44 | 121.3 | 16.0 |
| 45–54 | 126.5 | 15.6 |
| 55–64 | 132.4 | 18.5 |
| 65–74 | 134.9 | 16.3 |
| 75–84 | 136.5 | 16.0 |
| 85 years and over | 140.2 | 15.7 |
| Females | | |
| Age group | **Average SBP** | **Std Dev** |
| 18–24 | 107.6 | 16.5 |
| 25–34 | 108.5 | 17.1 |
| 35–44 | 112.5 | 16.2 |
| 45–54 | 119.6 | 20.1 |
| 55–64 | 126.8 | 19.0 |
| 65–74 | 133.6 | 16.7 |
| 75–84 | 137.9 | 14.8 |
| 85 years and over | 140.8 | 16.2 |

Source: Australian Bureau of Statistics. National Health Survey: First results, 2017–18. 2018. (25)

*Table 5. Relative risks from 10 mmHg increase in systolic blood pressure to ischemic heart disease and stroke*

|  | Ischemic heart disease | Ischemic stroke | Hemorrhagic stroke |
| --- | --- | --- | --- |
| Age groups | **RR (95%CI)** | **RR (95%CI)** | **RR (95%CI)** |
| 25-29 | 1.972 (1.436 to 2.598) | 1.854 (1.394 to 2.590) | 2.134 (1.554 to 2.919) |
| 30-34 | 1.818 (1.458 to 2.207) | 1.774 (1.426 to 2.253) | 2.050 (1.593 to 2.661) |
| 35-40 | 1.665 (1.458 to 1.911) | 1.694 (1.404 to 2.036) | 1.966 (1.588 to 2.465) |
| 40-45 | 1.568 (1.398 to 1.799) | 1.628 (1.353 to 1.950) | 1.874 (1.491 to 2.303) |
| 45-49 | 1.527 (1.393 to 1.706) | 1.574 (1.359 to 1.825) | 1.775 (1.481 to 2.117) |
| 50-54 | 1.487 (1.385 to 1.620) | 1.521 (1.360 to 1.700) | 1.676 (1.446 to 1.934) |
| 55-59 | 1.446 (1.368 to 1.536) | 1.468 (1.344 to 1.598) | 1.577 (1.399 to 1.755) |
| 60-64 | 1.405 (1.332 to 1.489) | 1.414 (1.301 to 1.524) | 1.478 (1.330 to 1.619) |
| 65-69 | 1.364 (1.255 to 1.456) | 1.361 (1.214 to 1.490) | 1.379 (1.207 to 1.540) |
| 70-74 | 1.330 (1.222 to 1.424) | 1.318 (1.168 to 1.452) | 1.323 (1.162 to 1.495) |
| 75-79 | 1.303 (1.225 to 1.404) | 1.284 (1.177 to 1.390) | 1.311 (1.192 to 1.450) |
| 80+ | 1.266 (1.133 to 1.437) | 1.201 (1.108 to 1.370) | 1.279 (1.126 to 1.519) |

Source: eTable 1. Forouzanfar MH, Liu P, Roth GA, Ng M, Biryukov S, Marczak L, et al. Global Burden of Hypertension and Systolic Blood Pressure of at Least 110 to 115 mm Hg, 1990-2015. (26)

Source of data on exposure

Prevalence of unhealthy indoor temperature in base year

Prevalence of indoor cold was obtained from the 2016 Australian Housing Conditions Dataset. Data was available for 4501 household from New South Wales, South Australia, and Victoria.

Trends in prevalence of unhealthy indoor temperature for projections

Disease trends: Annual percentage change for all-cause mortality rates

All-cause mortality rates by sex and 5-year age groups for Australia were obtained from GBD. For each sex, annual percentage changes (APCs) were estimated using Poisson regression. Interaction terms between age groups and year in separate models by sex, allowing for age variations in all-cause mortality rates.

Ln[all-cause mortality rate]= intercept + B1.Age[5yrcat] + B2.Year + B3.Age[1-19].Year + B4.Age[20-

34].Year + B5.Age[35-49].Year + B6.Age[50-79].Year + B7.Age[80+].Year

Where:

B1, B2, …, B7 are the model coefficients, with B1 actually being a vector of coefficients for all fiveyear

age categories.

Age[5yrcat] is the categorical variable for 5 year age groups

Year is calendar year as a continuous variable

Age[1-19], Age[20-34], …, Age[80+] are dummy variables for these age groups, used in interactions

with calendar year.

The coefficients B2 to B7 were then converted into APCs in mortality by age, and served as inputs to

the model.

**DISMOD coherence checks on epidemiological data from IHME**

Ischemic heart disease

Males

Females

Ischemic stroke

Males

Females

Haemorrhagic stroke

Males

Females

# References

1. Saeki K, Obayashi K, Kurumatani N. Short-term effects of instruction in home heating on indoor temperature and blood pressure in elderly people: a randomized controlled trial. J Hypertens. 2015;33(11):2338-43. Epub 2015/09/16.

2. Pierse N, Arnold R, Keall M, Howden-Chapman P, Crane J, Cunningham M, et al. Modelling the effects of low indoor temperatures on the lung function of children with asthma. J Epidemiol Community Health. 2013;67(11):918-25. Epub 2013/08/14.

3. Shiue I. Cold homes are associated with poor biomarkers and less blood pressure check-up: English Longitudinal Study of Ageing, 2012-2013. Environ Sci Pollut Res Int. 2016;23(7):7055-9. Epub 2016/02/14.

4. Mu Z, Chen PL, Geng FH, Ren L, Gu WC, Ma JY, et al. Synergistic effects of temperature and humidity on the symptoms of COPD patients. Int J Biometeorol. 2017;61(11):1919-25. Epub 2017/06/02.

5. Osman LM, Ayres JG, Garden C, Reglitz K, Lyon J, Douglas JG. Home warmth and health status of COPD patients. Eur J Public Health. 2008;18(4):399-405. Epub 2008/03/28.

6. Saeki K, Obayashi K, Iwamoto J, Tanaka Y, Tanaka N, Takata S, et al. Influence of room heating on ambulatory blood pressure in winter: a randomised controlled study. J Epidemiol Community Health. 2013;67(6):484-90. Epub 2013/03/01.

7. Bruce N, Elford J, Wannamethee G, Shaper AG. The contribution of environmental temperature and humidity to geographic variations in blood pressure. J Hypertens. 1991;9(9):851-8. Epub 1991/09/01.

8. Barnett AG, Sans S, Salomaa V, Kuulasmaa K, Dobson AJ, Project WM. The effect of temperature on systolic blood pressure. Blood pressure monitoring. 2007;12(3):195-203.

9. Zhao H, Jivraj S, Moody A. ‘My blood pressure is low today, do you have the heating on?’The association between indoor temperature and blood pressure. Journal of hypertension. 2019;37(3):504-12.

10. Saeki K, Obayashi K, Iwamoto J, Tone N, Okamoto N, Tomioka K, et al. Stronger association of indoor temperature than outdoor temperature with blood pressure in colder months. Journal of hypertension. 2014;32(8):1582-9.

11. Saeki K, Obayashi K, Iwamoto J, Tone N, Okamoto N, Tomioka K, et al. The relationship between indoor, outdoor and ambient temperatures and morning BP surges from inter-seasonally repeated measurements. Journal of human hypertension. 2014;28(8):482-8.

12. Woodhouse PR, Khaw KT, Plummer M. Seasonal variation of blood pressure and its relationship to ambient temperature in an elderly population. J Hypertens. 1993;11(11):1267-74. Epub 1993/11/01.

13. Ross A, Collins M, Sanders C. Upper respiratory tract infection in children, domestic temperatures, and humidity. J Epidemiol Community Health. 1990;44(2):142-6. Epub 1990/06/01.

14. Barnett AG, Sans S, Salomaa V, Kuulasmaa K, Dobson AJ, Project WM. The effect of temperature on systolic blood pressure. Blood Press Monit. 2007;12(3):195-203. Epub 2007/05/15.

15. Harrington L, Aye L, Fuller RJAQ, Change C. Characterising indoor air temperature and humidity in Australian homes. 2015;49(4):21.

16. Ambrose M, James M, Law A, Osman P, White SJCoA, Canberra. The evaluation of the 5-star energy efficiency standard for residential buildings. 2013.

17. Cheng M, Galbally I, Gillett R, Keywood M, Lawson S, Molloy S, et al. Indoor air project part 1: Main report. Indoor Air in Typical Australian Dwellings. 2010.

18. Soebarto V, Bennetts HJB, environment. Thermal comfort and occupant responses during summer in a low to middle income housing development in South Australia. 2014;75:19-29.

19. Asumadu-Sakyi AB, Barnett AG, Thai P, Jayaratne ER, Miller W, Thompson MH, et al. The relationship between indoor and outdoor temperature in warm and cool seasons in houses in Brisbane, Australia. Energy and Buildings. 2019;191:127-42.

20. Daniel L, Williamson T, Soebarto V, Chen D, editors. A study of thermal Mavericks in Australia. Proceedings of the 8th Windsor Conference: Counting the Cost of Comfort in a Changing World; 2014: Cumberland Lodge, Windsor, UK.

21. de Dear R, Kim J, Parkinson TJE, Buildings. Residential adaptive comfort in a humid subtropical climate—Sydney Australia. 2018;158:1296-305.

22. Haddad S, Pignatta G, Paolini R, Synnefa A, Santamouris M, editors. An extensive study on the relationship between energy use, indoor thermal comfort, and health in social housing: the case of the New South Wales, Australia. IOP Conference Series: Materials Science and Engineering; 2019: IOP Publishing.

23. Loughnan M, Carroll M, Tapper NJ. The relationship between housing and heat wave resilience in older people. International journal of biometeorology. 2015;59(9):1291-8.

24. Baker, Emma; Beer, Andrew; Zillante, George; London, Kerry; Bentley, Rebecca; Hulse, Kathleen; Pawson, Hal; Randolph, Bill; Stone, Wendy; Rajagopolan, Priya, 2019, "The Australian Housing Conditions Dataset", doi:10.26193/RDMRD3, ADA Dataverse, V1

25. Australian Bureau of Statistics. National Health Survey: First results, 2017–18. . 2018.

26. Forouzanfar MH, Liu P, Roth GA, Ng M, Biryukov S, Marczak L, et al. Global Burden of Hypertension and Systolic Blood Pressure of at Least 110 to 115 mm Hg, 1990-2015. JAMA. 2017;317(2):165-82. Epub 2017/01/18.
